# HIF-1α Reduction by Lowering Intraocular Pressure Alleviated Retinal Neovascularization

**DOI:** 10.3390/biom13101532

**Published:** 2023-10-17

**Authors:** Ziqi Yang, Biyan Ni, Tian Zhou, Zijing Huang, Hong Zhou, Yang Zhou, Shiya Lin, Chang He, Xialin Liu

**Affiliations:** 1 State Key Laboratory of Ophthalmology, Zhongshan Ophthalmic Center, Sun Yat-sen University, Guangdong Provincial Key Laboratory of Ophthalmology and Visual Science, Guangzhou 510060, China; yangziqi@gzzoc.com (Z.Y.); nibiyan@gzzoc.com (B.N.); zhoutian@gzzoc.com (T.Z.); huangzj@jsiec.org (Z.H.); zhouh256@mail2.sysu.edu.cn (H.Z.); zhouyang@gzzoc.com (Y.Z.); linshiya@gzzoc.com (S.L.); 2 Joint Shantou International Eye Center of Shantou University and The Chinese University of Hong Kong, Shantou 515041, China

**Keywords:** hypoxia-inducible factor-1 alpha (HIF-1α), intraocular pressure (IOP), retinal neovascularization, oxygen-induced retinopathy (OIR), intravitreal injection

## Abstract

Hypoxia-induced retinal neovascularization is a leading cause of blindness worldwide. Oxygen-induced retinopathy (OIR) mouse, a well-established angiogenesis model, has been extensively used to evaluate the effect of anti-angiogenic agents through intravitreal injection. Here, we serendipitously found that the needles used for intravitreal injection caused an unexpected “anti-angiogenic” effect in the OIR mice. To evaluate the effects of various intravitreal puncture sizes on retinal neovascularization and explore the potential underlying mechanism, intravitreal punctures using 0.5 mm (25 G), 0.3 mm (30 G), or 0.21 mm (33 G) needles were performed in OIR mice. Compared with 0.3 mm and 0.21 mm puncture, the 0.5 mm puncture remarkably suppressed the formation of pathological angiogenesis, inhibited vascular leakage, and remodeled the retinal vasculature. Mechanistically, the 0.5 mm puncture induced a substantial reduction in intraocular pressure (IOP), leading to an improvement in oxygen partial pressure (pO_2_) and significant reduction in Hif1a expression, resulting in resolution of angiogenic and inflammatory responses. Furthermore, IOP-lowering drugs, Travatan or Azarga, also promoted the alleviation of hypoxia and exhibited a potent anti-angiogenesis efficacy. Our study revealed an acute and significant reduction in IOP caused by a large puncture, which could remarkably suppress HIF-1α-mediated retinal neovascularization, indicating that lowering IOP may be a promising therapeutic avenue for treating retinal neovascular diseases.

## 1. Introduction

Pathological neovascularization is a common feature of various retinal diseases, including retinopathy of prematurity (ROP), proliferative diabetic retinopathy (PDR), retinal vein occlusion (RVO), wet age-related macular degeneration (wet AMD), etc. [1]. It causes retinal edema, hemorrhage, inflammation, and fibrotic scarring, leading to severe visual impairment and even blindness [2,3]. Despite variations in the underlying causes and risk factors, hypoxia-induced production of angiogenic mediators such as vascular endothelial growth factor (VEGF) is widely recognized to play a critical role in triggering the formation of new blood vessels [4]. In the clinic, several anti-VEGF drugs and retinal laser photocoagulation techniques have been applied to reduce retinal hypoxia, consequently leading to the inhibition of retinal angiogenesis. However, there remains limitations such as resistance to anti-VEGF drugs and irreversible peripheral visual function damage caused by laser photocoagulation [5,6]. Therefore, alternative approaches that target the hypoxia-induced angiogenesis are actively being explored.

Hypoxia-inducible factor-1α (HIF-1α), a crucial transcription factor that forms a heterodimer with HIF-1β and binds to hypoxia-responsive elements (HREs) within target gene promoters, plays a pivotal role in cellular responses to hypoxia. HIF-1α has been recognized as inducing the expression of angiogenic factors such as VEGF, promoting the formation of new blood vessels. In addition, HIF-1α activation in response to hypoxia insult drives metabolic adaptations, to ensure immune cell activation and effective responses, orchestrating the inflammatory environment for retinal angiogenesis. Targeting HIF-1α signaling seems to be a promising approach for regulating hypoxia-induced angiogenesis.

Oxygen-induced retinopathy (OIR) is the most widely used model for retinal neovascular diseases, such as ROP, PDR, and RVO [4,7,8]. In the application of this hypoxia-induced angiogenesis model, it is common to perform intravitreal injection of various agents, to elucidate the molecular mechanisms involved in retinal neovascularization or to test the efficacy of new potential anti-angiogenic agents. When performing intravitreal injection, a Hamilton microliter syringe is usually used to inject 0.5–2 μL reagent in an OIR model. In some cases, the needle of the microliter syringe is very soft and may become dull after multiple injections, making it difficult to penetrate the eyeball effectively. Therefore, some researches chose sharp and disposable needles to pre-create a puncture channel, followed by using the microliter syringe to inject the corresponding volume of reagents [9,10]. In this study, we serendipitously found that a 0.5 mm puncture (using a 25 G needle) without delivering any reagents could almost completely inhibit the formation of pathological angiogenesis in the OIR model, while a 0.3 mm puncture (using a 30 G needle) or 0.21 mm direct injection procedure (using 33 G needle) did not influence the development of neovascularization.

Compared with the 0.3 mm or 0.21 mm puncture group, the 0.5 mm puncture apparently caused the leakage of vitreous fluid, concomitant with a collapsed eyeball, indicating a sudden drop in intraocular pressure (IOP) induced by the 0.5 mm puncture. As we know, ocular perfusion pressure, a vital factor influencing ocular blood flow, is obviously affected by changes in IOP [11]. Elevated IOP can hinder the blood flow and diminish the perfusion pressure within the retinal vasculature, leading to insufficient oxygen delivery and subsequent retinal hypoxia [12,13]. Remarkably, 0.5 mm puncture induced a substantial decrease in IOP, subsequently elevating ocular perfusion pressure and retinal oxygen partial pressure (pO_2_). These alterations hold the potential to reduce HIF-1α expression and mitigate the pathological angiogenesis. In addition, the strikingly contrary outcomes observed with varying needle sizes suggested that large size needles (≥0.5 mm) for intravitreal injection should be avoided in the OIR model, due to the potential role of IOP reduction in inhibiting HIF-1α-mediated retinal neovascularization.

## 2. Materials and Methods

### 2.1. Mice for OIR Model and Needle Puncture

C57BL/6J mice were obtained from the Animal Center of Guangzhou University of Chinese Medicine and were housed in a specific pathogen-free facility in the Animal Laboratories of Zhongshan Ophthalmic Center. All animal experiments were approved by Institutional Animal Care and Use Committee of Zhongshan Ophthalmic Center, Sun Yat-sen University (Ethic number: Z2022042, approved on 14 June 2022). The OIR model was established as described previously [7]. Newborn mice and their nursing mothers were exposed to 75% O_2_ in an oxygen chamber from postnatal day 7 (P7) to P12. These mice were randomized into groups and received intravitreal puncture using needles of different calibers immediately upon returning to room air environment on P12. A disposable sharp needle (25 G with a 0.5 mm needle outer diameter, BD/30 G with a 0.3 mm needle outer diameter, BD) was inserted vertically into the eye wall 1 mm posterior to the limbus. The resulting scleral tunnel could be easily accessed for the 33 G microliter syringe injection (a 0.21 mm needle outer diameter, Hamilton). Alternatively, the 33 G microliter syringe needle could be directly inserted vertically into the eye wall. Pups weighing more than 6 g were euthanized using carbon dioxide asphyxiation on P17 for further experimental analysis.

### 2.2. Measurement of pO_2_

The measurement of particle oxygen pressure was performed as previously described [14]. Pups were anesthetized through intraperitoneal injection of 50 mg/kg pentobarbital sodium. An incision was made in the lateral and temporal eyelid to expose the eye ball, and then mice were positioned in a robotic stereotaxic apparatus. To measure oxygen levels, a custom-made oxygen sensitive microelectrode for mice was inserted into the vitreous through the limbus. The electrode’s position inside the eye was visualized using a contact lens and an operating microscope. The oxygen pressure was recorded until the values stabilized.

### 2.3. Measurement and Management of IOP

Pups were anesthetized using an intraperitoneal injection of 50 mg/kg pentobarbital sodium. IOP was measured daily at 10 AM using a rebound tonometer (iCare TONOLAB, Vantaa, Finland) from P12 to P17. At P12, as the mice received intravitreal puncture using needles immediately upon returning to the room air at 6 AM, two additional IOP measurements were taken, both before and after the puncture. Six consecutive probe-to-cornea contact measurements were averaged as one record. For lowering IOP, Travatan (0.004% travoprost, Novartis) and Azarga (10 mg/mL Brinzolamide + 5 mg/mL Timolol, Novartis) eye drops were applied to one eye at a dose of 5 µL from P12 to P17 once a day, while 5 µL of PBS eye drops were applied to the other eye as a control in the same pup mouse. IOP was measured before and 2 h after drug administration at P12. Subsequently, IOP was measured daily at 10 AM from P13 to P17.

### 2.4. H&E and Immunohistochemistry Staining

Eyes were fixed with 4% formalin overnight, embedded in paraffin, and cut into 5 μm vertical slices. Sections were then washed and treated with hematoxylin buffer for 10 min at room temperature. After rinsing in deionized water and dipping in 1% eosin solution for 15 s, sections were rehydrated in an alcohol gradient, rewashed, and mounted. The neovascular cell nuclei above the internal limiting membrane (ILM) nuclei were counted, and the mean of the 10 counted sections yielded the average neovascular cell nuclei per eye. At least six eyes were used for analysis. For immunohistochemistry staining, sections were subjected to antigen retrieval, then blocked with normal serum, and incubated overnight with diluted primary antibodies at 4 °C. The following primary antibodies were used: polyclonal rabbit anti-TGFb (Abcam, Cambridge, UK) and monoclonal rabbit anti-collagen III (Abcam). Normal IgG was used as a negative control. The secondary antibody Goat anti-Rabbit IgG (H+L) Cross-Adsorbed Secondary Antibody, HRP (Invitrogen, Carlsbad, CA, USA) was incubated for 2 h. Retinal histological changes were observed under a microscope (Leica DM4000, Wetzlar, Germany).

### 2.5. Immunofluorescence Staining of Retinal Wholemounts

Eyes were enucleated and fixed in 4% paraformaldehyde (PFA) fixative for 1 h and the retinae were removed carefully. The retinal whole-mounts were stained with Isolectin B4 (IB4, 1:50, Invitrogen) for 2 h at room temperature for quantification of neovascularization. For other immunofluorescence stainings, the retinae were incubated with 5% Bovine Serum Albumin (BSA) at 4 °C overnight for blocking and incubated with primary and secondary antibodies. The primary antibodies include anti-CD31 antibody (Merck Millipore, Darmstadt, Germany), anti-NG2 antibody (Servicebio, Wuhan, Hubei, China), anti-α-SMA antibody (Invitrogen), and anti-IBA1 antibody (Wako, Tokyo, Japan). The secondary antibodies included donkey anti-rabbit IgG H&L (Alexa Fluor 555) secondary antibody, goat anti-Armenian hamster IgG H&L (Alexa Fluor 488), and goat anti-mouse IgG H&L (Alexa Fluor 647) (Abcam). The retinae were washed extensively, flat-mounted, and observed using a confocal microscope (Carl Zeiss, Jena, Germany). Areas of retinal neovascularization and the avascular region were analyzed using Image-Pro Plus 6.0 (Media Cybernetic, Rockville, MD, USA).

### 2.6. Visualization of Retinal Vascular Permeability

Retinal vascular permeability was visualized through the leakage of Evans blue (EB). EB powder (Sigma, Darmstadt, Germany) was dissolved in PBS to make 2% EB solution and then intravenously injected at a dosage of 150 μg/kg. After 2 h, the mice were sacrificed, and their eyes were perfused with 4% PFA. Retinae were dissected, flat-mounted, and examined through fluorescence microscopy (Carl Zeiss, Jena, Germany).

### 2.7. Hypoxyprobe Staining

The hypoxic state in the retina was evaluated using a Hypoxyprobe RedAPC Kit, as previously described [15]. OIR mice at P12 or P17 were injected with Hypoxyprobe intraperitoneally at 2.5 mg per pup 1 h before they were sacrificed. Retinas were isolated after fixation of eyeballs in 4% PFA for 1 h, washed 3 times with PBST, blocked with 5% BSA for 1 h, and incubated with RED APC dye -MAb1 diluted with PBST at 4 °C overnight. Retinae were washed extensively and cut into four pieces before being flat-mounted on slides. All retinae were photographed using a confocal microscope (Carl Zeiss, Jena, Germany).

### 2.8. Real-Time Quantitative PCR Analysis

Total RNA was extracted from the retinae on ice using TRIzol (Invitrogen), and cDNA synthesis was performed using a Reverse Transcriptase Superscript II Kit (Invitrogen) according to the manufacturer’s instructions. Briefly, qPCR was performed in a total volume of 20 µL mixture containing 2 µL of cDNA, 10 µL of 2 × SYBR Premix Ex Taq, and 10 µmol/L of the primer pairs. GAPDH was used as a reference gene. Samples were incubated at 95 °C for 30 s and up to 40 cycles of 95 °C for 5 s and 60 °C for 34 s according to the protocol. The real-time PCR reaction was performed using a StepOnePlus™ real-time PCR instrument. The experiments were repeated independently three or more times.

### 2.9. Statistics

Representative images were displayed in figures, and the results were repeated at least 3 times. All data were statistically analyzed and presented as the mean ± standard error of the mean (SEM). Comparisons of three or four groups were analyzed using one-way ANOVA, and comparisons between two groups were performed using a two-tailed Student *t*-test. A value of *p* < 0.05 was considered statistically significant (* *p* < 0.05; ** *p* < 0.01; *** *p* < 0.001).

## 3. Results

### 3.1. Intravitreal Puncture of 0.5 mm Diameter Alleviated Pathological Angiogenesis and Remodeled Retinal Vasculature in the OIR Model

**Figure 1:**
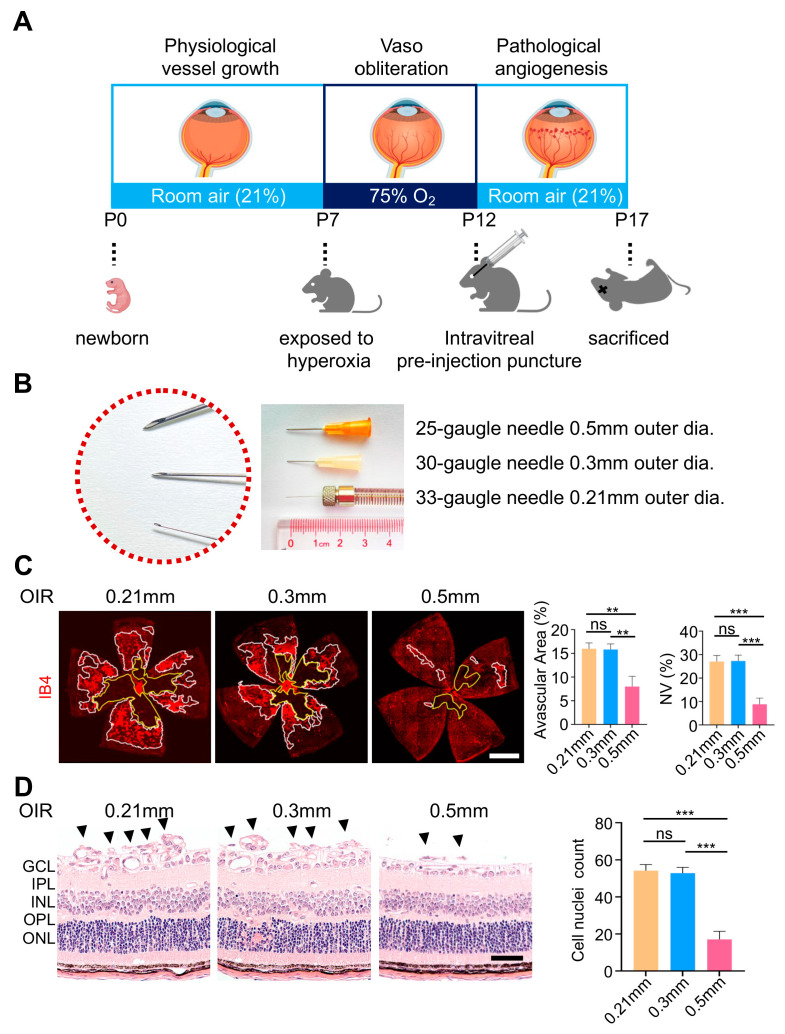
An intravitreal puncture of 0.5 mm diameter suppressed pathological angiogenesis in the oxygen-induced retinopathy (OIR) model. (**A**) Schematic of the mouse OIR model. After being exposed to a hyperoxic condition (75% O_2_) from P7 to P12, the mice were returned to room air (21% O_2_), and the relative hypoxia triggered the development of pathological angiogenesis peaking on P17. Intravitreal pre-injection puncture was usually performed on P12. (**B**) Needles used in this study. (**C**) Representative whole-mount images of IB4 immunofluorescence staining on P17. The 0.5 mm puncture significantly reduced the avascular area (yellow line) and new vascular tufts area (white line) compared with the 0.3 mm or 0.21 mm puncture groups. Scale bars: 1 mm. (**D**) Neovascular cellular nuclei anterior to the ILM (black arrowheads) were remarkably decreased after 0.5 mm puncture. ILM, internal limiting membrane; GCL, ganglion cell layer; IPL, inner plexiform layer; INL, inner nuclear layer; OPL, outer plexiform layer; ONL, outer nuclear layer. Scale bars: 50 µm. (n = 6 eyes mean  ±  SEM; ns, no significance; ** *p* < 0.01; *** *p* < 0.001).

The establishment of the OIR model is sketched in Figure 1A. Briefly, 7-day-old pups were exposed to a high oxygen condition (75%) for five days, followed by returning them to room air, to create a relative hypoxic state on P12. Then, the relative hypoxia triggered the abnormal formation of neovascular tufts, with the peak of angiogenesis on P17, when mice were sacrificed to evaluate neovascularization [7]. In particular, when applying OIR mice to test anti-angiogenic agents, drugs were usually injected intravitreally after performing the pre-injection puncture at P12. Figure 1B shows the commonly used needles with a size of 25 G or 30 G (0.5 mm or 0.3 mm in outer diameter) and a 33 G microliter syringe (0.21 mm in outer diameter) for intravitreal pre-injection puncture. Surprisingly, 0.5 mm puncture for intravitreal injection of different agents, either various therapeutic drugs or PBS/IgG controls, showed similar effects on angiogenesis formation (data not shown). Therefore, we performed intravitreal punctures without any drug injection and compared the effects of 0.5 mm, 0.3 mm, and 0.21 mm punctures on retinal neovascularization in the OIR mice. Unexpectedly, the 0.5 mm puncture on P12 significantly reduced the avascular area of the OIR retinae and suppressed the development of neovascular tufts on P17 in comparison to 0.3 mm or 0.21 mm puncture (Figure 1C). In addition, the number of cellular nuclei penetrating the ILM in HE sections was also remarkably reduced in the 0.5 mm group (Figure 1D), further indicating that 0.5 mm puncture itself could suppress the formation of retinal neovascularization in the OIR model. Interestingly, the normal retinal vasculature remained largely undisturbed after 0.5 mm, 0.3 mm, or 0.21 mm puncture (Figure S1).

**Figure 2:**
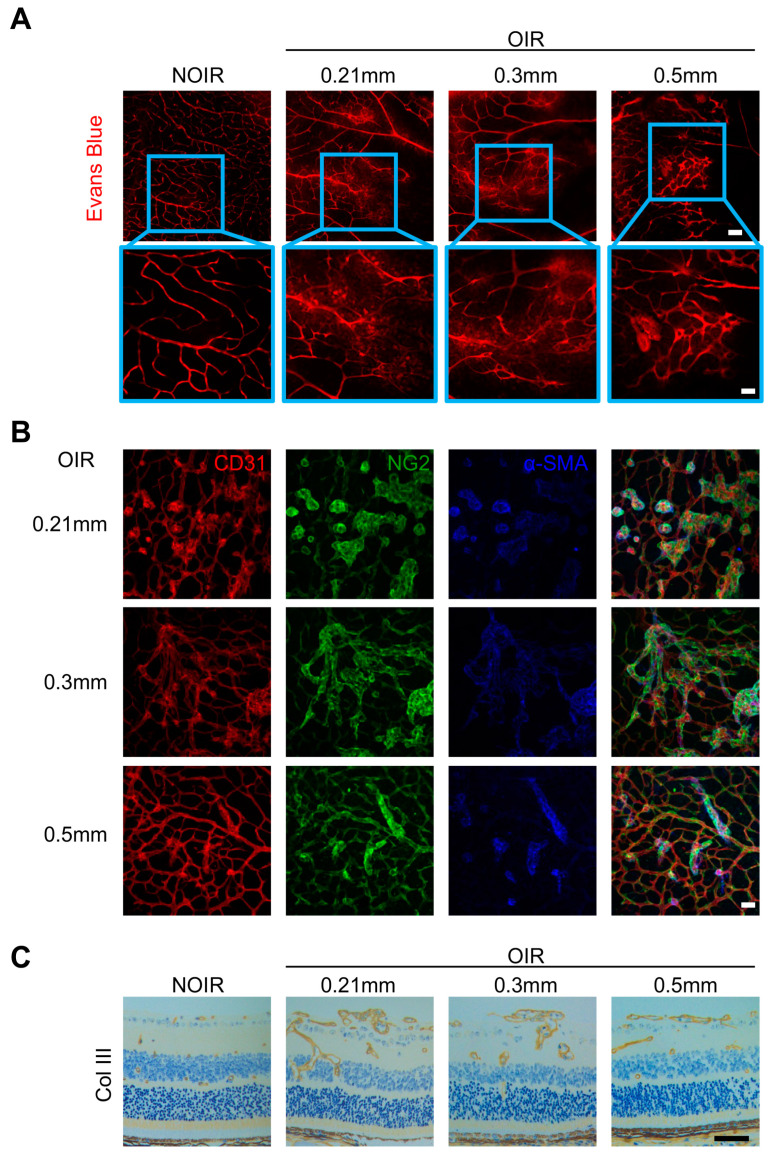
The 0.5 mm intravitreal puncture alleviated leakage and abnormal collagen accumulation in the retinal vasculature. (**A**) Representative images of Evans blue fluorescence staining. Less Evans blue diffusion was observed in the 0.5 mm group. Scale bars: 100 µm (top); 50 µm (bottom). (**B**) The retinal vasculature is displayed with CD31^+^ endothelial cells and NG2^+^α-SMA^+^ mural cells. Disarranged angiogenic sprouts were observed in punctures of 0.21 mm and 0.3 mm group. In contrast, 0.5 mm puncture presented a relative regular and organized vasculature. Scale bars: 50 µm. (**C**) Representative collagen III staining images of retinal sections on P17 showed less collagen accumulation around the retinal vasculature in the 0.5 mm puncture group. Scale bars: 50 µm.

In order to further evaluate the retinal vasculature after puncture, vascular permeability was determined using Evans blue staining. As shown in the Figure 2A, the leakage of the neovascular tufts in OIR retinae was significantly alleviated by 0.5 mm puncture, while the 0.3 mm and 0.21 mm group still presented obvious vascular leakage. Both the 0.3 mm and 0.21 mm groups presented abnormal and irregular retinal vasculature with CD31^+^ endothelial cells and NG2^+^α-SMA^+^ mural cells. In contrast, the 0.5 mm puncture induced a significant restoration of retinal vasculature, with an organized CD31^+^ and NG2^+^α-SMA^+^ network (Figure 2B). Collagen staining also revealed that the 0.5 mm puncture eliminated abnormal collagen accumulation around the neovascular tufts (Figure 2C). Together, 0.5 mm puncture significantly inhibited the formation of pathological angiogenesis and improved the vasculature remodeling.

### 3.2. The 0.5 mm Intravitreal Puncture Prevented Retinal Hypoxia in OIR Retinae

**Figure 3:**
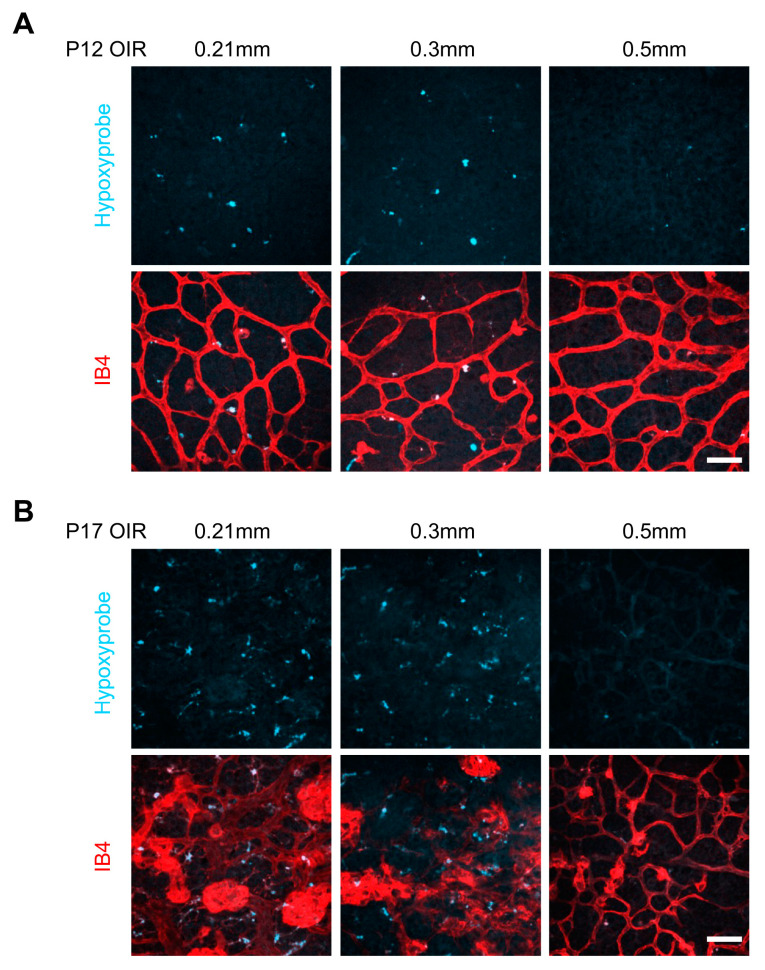
The 0.5 mm intravitreal puncture significantly alleviated hypoxia in the OIR retina. (**A**,**B**) Representative retinal whole-mount images of hypoxyprobe staining. The fluorescence intensity of the hypoxyprobe after 0.5 mm puncture significantly decreased on P12 (**A**) and P17 (**B**) compared with that after 0.3 mm puncture and 0.21 mm puncture. Scale bars: 50 µm.

As hypoxia is recognized as the pure inducer of retinal angiogenesis in OIR [16], we applied a sensitive detection method (the Hypoxyprobe, a hypoxia molecular probe) to determine the retinal hypoxic state. Remarkably, the retinal flat-mounting showed the presence of hypoxia in OIR retinae on P12. The 0.3 mm puncture and 0.21 mm puncture groups exhibited a similar level of hypoxyprobe^+^ staining, while notably less hypoxyprobe signaling in OIR retinae was observed in the 0.5 mm puncture group, indicating that the 0.5 mm puncture induced a rapid relief of hypoxia state on P12 (Figure 3A). In addition, the hypoxia condition became worse with upregulated hypoxyprobe signaling, probably due to the functionless neovasculature on P17 (Figure 3B). However, the 0.5 mm puncture presented much less hypoxyprobe signaling (Figure 3B), indicating the alleviation of retinal hypoxia after 0.5 mm puncture.

### 3.3. The 0.5 mm Puncture Induced a Substantial Reduction in IOP and Improvement in Retinal Oxygenation In Vivo

**Figure 4:**
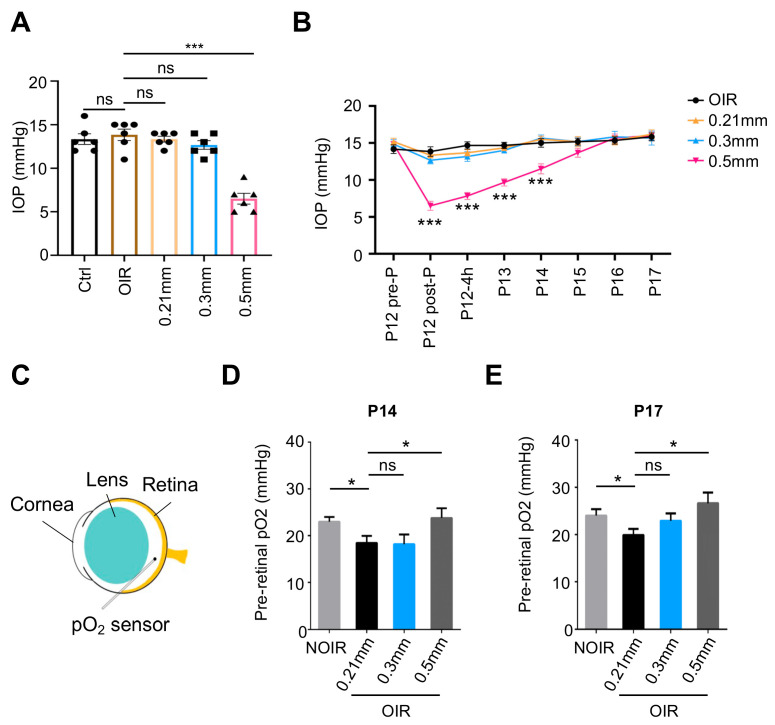
The 0.5 mm puncture induced a substantial reduction in intraocular pressure (IOP) and an improvement in retinal oxygen partial pressure (pO_2_). (**A**) The 0.5 mm puncture on P12 significantly reduced IOP, while 0.3 mm puncture made no significant changes to IOP (n = 6 eyes, ns, no significance; *** *p* < 0.001). (**B**) Dynamic changes in IOP during P12 to P17. After 0.5 mm puncture on P12, IOP was sharply decreased and sustained a relative lower level until P14 compared with 0.3 mm puncture and 0.21 mm puncture in the OIR model (n = 6 eyes, *** *p* < 0.001). (**C**) Schematic showing the pO_2_ measurement in the epiretinal space of mice. After 0.5 mm puncture, the pO_2_ significantly increased at P14 (**D**) and P17 (**E**) (n = 6 eyes, ns, no significance; * *p* < 0.05).

To further investigate the underlying mechanism of hypoxia reduction after 0.5 mm puncture, we examined the IOP, since the 0.5 mm puncture caused a leakage of vitreous fluid. Here, we found that 0.5 mm puncture resulted in a substantial decrease in IOP, to approximately 45% of its baseline level, while punctures of 0.3 mm and 0.21 mm did not elicit a statistically significant change in IOP (Figure 4A). Dynamic monitoring of IOP showed that the IOP remained consistently lower after a 0.5 mm puncture during the subsequent 2 days, coinciding with a period of severe retinal hypoxia (Figure 4B). Oxygen partial pressure, an in vivo parameter indicative of ocular hypoxia, has been shown to be negatively related with IOP [17]. Then, pO_2_ was detected using an electronic oxygen sensor positioned in the epiretinal space in vivo (Figure 4C). The results showed that pO_2_ in the epiretinal space of OIR mice in the 0.21 mm group on both P14 and P17 was lower than that of NOIR mice, indicative of a hypoxic state in the intravitreally injected OIR retinae (Figure 4D,E). While the pO_2_ in the 0.3 mm puncture group had no significant difference in comparison to the 0.21 mm puncture group, the retinal pO_2_ after 0.5 mm puncture recovered to a normal level, indicating that the 0.5 mm puncture induced a remarkable recovery of retinal hypoxia (Figure 4D,E). Together, the 0.5 mm puncture induced a significant reduction in IOP, leading to the mitigation of retinal hypoxia.

### 3.4. The 0.5 mm Puncture Suppressed the Expressions of Hif1a and Relevant Angiogenic-Inflammatory Genes in Retina

**Figure 5:**
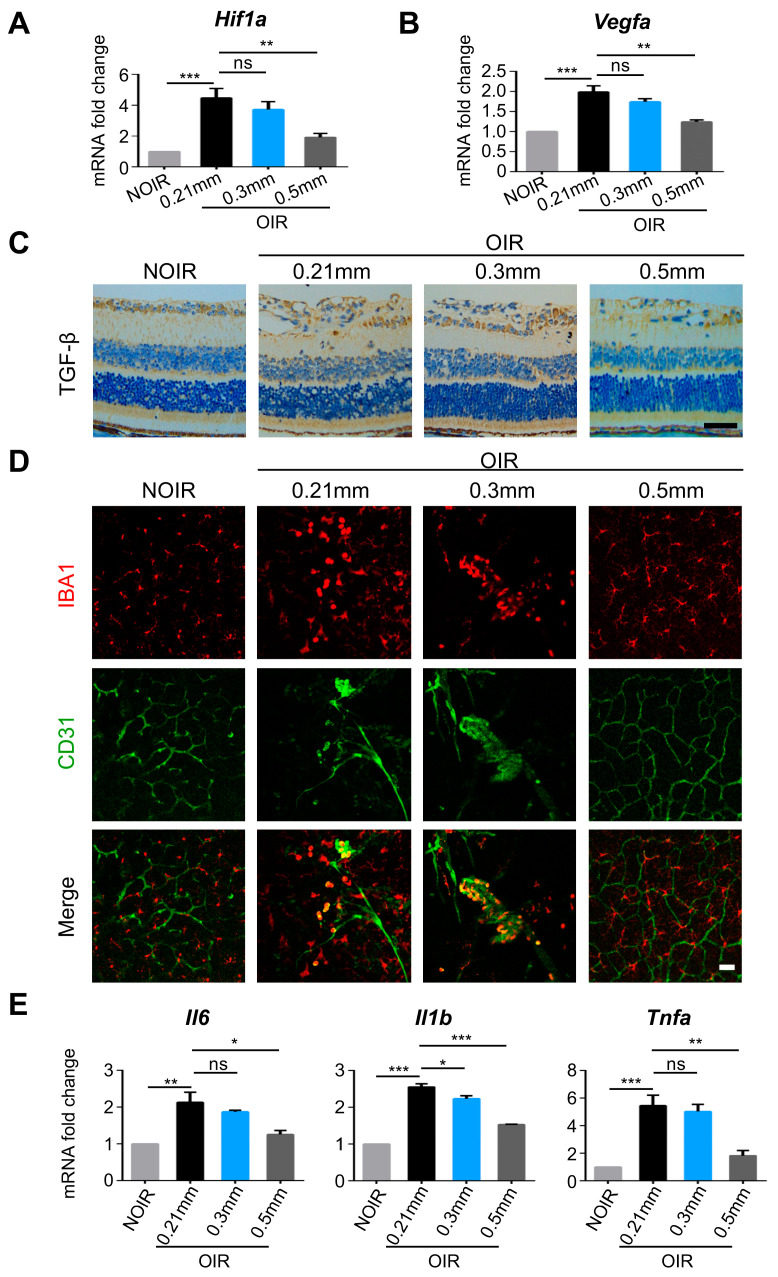
The 0.5 mm puncture suppressed the expressions of *Hif1a* and relevant angiogenic-inflammatory genes in retina. (**A**,**B**) *Hif1a* and *Vegfa* were increased in the OIR retina, which were abrogated by 0.5 mm puncture (n = 6 retinae, ns, no significance; ** *p* < 0.01; *** *p* < 0.001). (**C**) The protein level of TGF-β was significantly reduced after 0.5 mm puncture. Scale bars: 50 µm. (**D**) The IBA1^+^ microglia were activated and recruited to perivascular locations in OIR retinae with 0.21 mm and 0.3 mm punctures, which were restored after 0.5 mm puncture. Scale bars: 50 µm. (**E**) The mRNA expressions of *Il6*, *Il1b,* and *Tnfa* were decreased by 0.5 mm puncture (n = 6 retinae, ns, no significance; * *p* < 0.05; ** *p* < 0.01; *** *p* < 0.001).

HIF-1α is an essential component in pathways that govern cellular response under hypoxia and regulate immune cell activation [18]. As shown in Figure 5A, the expression of *Hif1a* was decreased after 0.5 mm puncture. Consequently, the expression of pro-angiogenic factor *Vegfa* was reduced significantly (Figure 5B). The protein expression of TGF-β, closely associated with neovascularization [19,20], was also eliminated after 0.5 mm puncture (Figure 5C). In addition, we detected the activation of microglia, the hallmark of retinal inflammation, that contributed to retinal angiogenesis [21]. Interestingly, the activated microglia in OIR were distributed around the neovascular tufts (Figure 5D). In contrast, the microglia in OIR after 0.5 mm puncture displayed a delicate and highly branched appearance, indicating less activation of microglia (Figure 5D). qPCR showed that pro-inflammatory factors including *Il6*, *Il1b*, and *Tnfa* were also decreased after 0.5 mm puncture, indicating that the inflammation was suppressed by 0.5 mm puncture (Figure 5E). Together, these results suggested that the 0.5 mm puncture prevented the upregulation of *Hif1a* and facilitated a significant relief of the pro-angiogenic and pro-inflammatory microenvironment in the OIR retina.

### 3.5. IOP-Lowering Drugs Attenuated Hypoxia-Induced Angiogenesis in the OIR Retina

**Figure 6:**
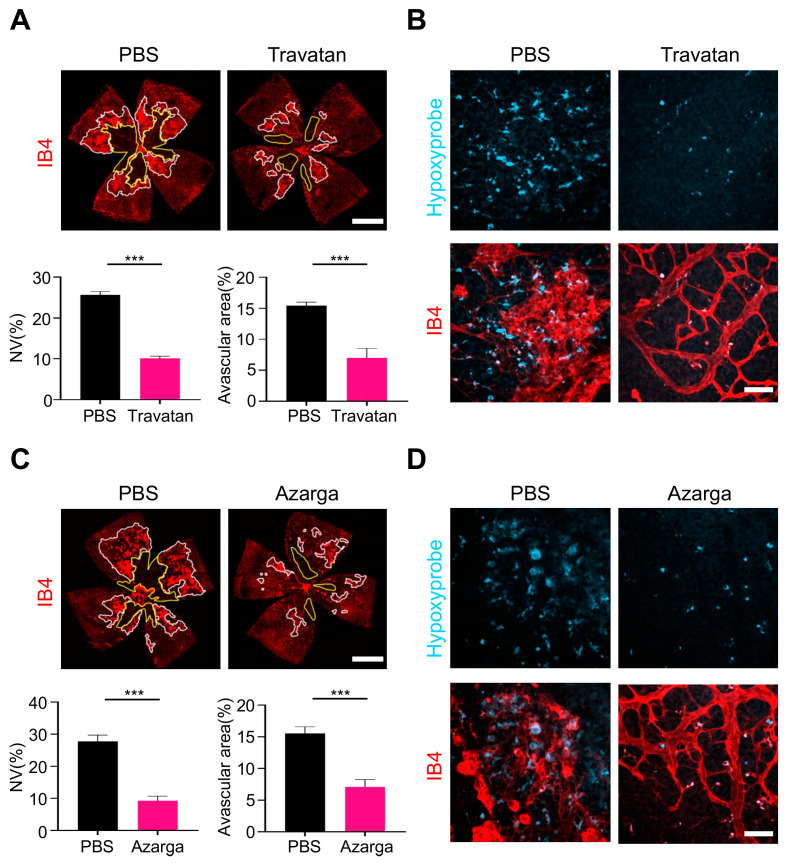
IOP- lowering drugs attenuated retinal neovascularization in the OIR model. (**A**) Travatan eye drop suppressed the formation of angiogenesis by over 60% and reduced avascular area in OIR. Scale bars: 1 mm. (**B**) Representative images of hypoxyprobe immunofluorescence of retinal flat-mount after Travatan treatment. Travatan alleviated the hypoxic state of retinae. Scale bars: 50 µm. (**C**) Azarga eye drop reduced the neovascular area by about 60% compared to the neovascularization of the PBS eye drop control. Avascular area was also reduced by 50% after administration of Azarga eye drop. Scale bars: 1 mm. (n = 6 eyes, mean ± SEM; *** *p* < 0.001) (**D**) Hypoxyprobe immunofluorescence of retinal flat-mount showing the alleviation of hypoxia after Azarga eye drop treatment. Scale bars: 50 µm.

Finally, we evaluated the anti-angiogenic effects of lowering IOP drugs, to further confirm the relationship between IOP and angiogenesis. Travatan, a commonly used Prostaglandin analogue (PGAs) drug containing 0.004% travoprost, and Azarga, another combination medication containing brinzolamide and timolol maleate, were used. Both Travatan and Azarga reduced the IOP as early as 2 h after treatment and maintained the effect for 5 days (Figure S2A,B). Remarkably, five-day usage of Travatan could suppress the pathological neovascularization to an extent to 60% (Figure 6A). In addition, the avascular area was also significantly reduced in the IB4-staining wholemounts (Figure 6A), and the hypoxyprobe results showed an obvious reduction in retinal hypoxia after Travatan usage (Figure 6B), indicating the close relationship between retinal hypoxia relief and IOP reduction. Similarly, Azarga could also alleviate the hypoxia with reduced avascular area and less hypoxyprobe signaling, leading to angiogenesis area inhibition by about 60% (Figure 6C,D). Together, the IOP-lowering drugs could alleviate retinal hypoxia and further suppress retinal neovascularization.

## 4. Discussion

In this study, we surprisingly found that large needle puncture (0.5 mm) for intravitreal injection caused an unexpected “anti-angiogenic” effect in OIR mice, indicating that an acute and significant reduction in IOP had the potential to counteract the hypoxia-induced angiogenesis. The 0.5 mm puncture induced a reduction in IOP and an improvement in pO_2_ and enhancement in oxygen delivery to the retina. Consequently, this ameliorated the hypoxic stimulus, leading to a decrease in the expression of Hif1a and angiogenic factors, thereby inhibiting pathological angiogenesis in the retina. This is the first study to report that IOP reduction achieved through intravitreal puncture can elicit such a significant anti-angiogenesis effect.

As we know, elevated IOP in glaucoma is associated with impaired blood flow to the retina and optic nerves, resulting in ischemic and hypoxic damage to the neuroretinal tissue [22,23]. Lowering IOP has proven beneficial in alleviating the mechanical compression and vascular compromise that occur in glaucoma. Clinically, it has been observed that patients with neovascular glaucoma experience significant regression of new blood vessels after the glaucoma surgery for lowering IOP. In addition, studies have indicated that lowering IOP can increase choroidal blood perfusion and alleviate scleral hypoxia, suggesting its potential role in managing high myopia progression. In patients with wet AMD receiving anti-VEGF therapy, simultaneous IOP reduction has been reported to contribute to improved visual outcomes after 2 years [24]. However, direct evidence demonstrating the notion that lowering IOP can prevent neovascularization is limited and the underlying mechanisms are not fully understood. In this study, use of a 25 G needle (0.5 mm) for intravitreal injection induced a sharp decline of IOP and probably enhanced the retinal perfusion and improved the blood supply to the retina, leading to alleviation of retinal hypoxia. Hypoxia, a recognized potent trigger, promotes the activation of Hif1a transcription factor, which subsequently instigates the expression of pro-angiogenic genes, including *Vegfa* and other angiogenic factors. Such processes lead to the development of pathological neovascularization [25]. Here, the IOP reduction achieved through intravitreal puncture downregulated the expression of *Hif1a* and *Vegfa*. This regulatory effect ultimately suppressed hypoxia-induced angiogenesis in the retina.

We have further proposed the therapeutic concept that IOP-lowering drugs may serve as a potential therapeutic strategy for managing retinal neovascular diseases. We detected the anti-angiogenesis effects of Azarga and Travatan, two commonly prescribed IOP-lowering medications in clinical practice. Despite Travatan lowering IOP by promoting aqueous humor outflow, and Azarga reducing IOP by decreasing aqueous humor production, both drugs achieve a similar level of IOP reduction and alleviation of neovascularization in OIR mice, indicating these non-invasive interventions aimed at lowering IOP could have potential for the treatment of retinal neovascular diseases. However, the direct vascular impact of these drugs could not be ruled out. Previous studies have shown that prostaglandin analogue, the main component of Travatan, could induce vessel vasodilation and increase capillary permeability [26]. Blockers of carbonic anhydrase, similarly to Azarga, may relax pericytes and improve the retinal blood supply by increasing the pH difference between the intracellular and extracellular space [27]. Therefore, the responses to IOP-lowering drugs are variable and individual, and further research and clinical studies are needed to fully understand the extent of their efficacy and applicability in managing retinal neovascular diseases.

The OIR mouse, the most commonly used and classical retinal neovascularization model, is applied to investigate the pathogenesis of retinal neovascularization and evaluate the efficacy of various agents in inhibiting neovascularization. In many cases, the agents need to be injected into the vitreous cavity and standardization of the intravitreal injection procedure directly affects the research results. Therefore, there is an urgent need to standardize the intravitreal injection procedure, to optimize the use of the OIR model. Our study found that using a 0.5 mm 25 G needle for intravitreal injection caused unexpected “anti-angiogenic” effects, while a 0.3 mm 30 G needle had no corresponding effect on angiogenesis and nor did a typical 0.21 mm Hamilton 33 G needle. This suggested that attention should be paid to the needle size during the intravitreal injection procedure in mice, to avoid confusing results caused by IOP decrease. It is advisable to avoid using needle diameters larger than 0.3 mm, as this may cause fluctuations in IOP, potentially affecting the outcome of neovascularization in the OIR model. In addition, it is worth noting that the anatomy of the mouse and human eyes is very different. It is indeed possible that a 0.5 mm intravitreal puncture on human eyes may not lead to a significant IOP reduction as observed in mice.

In summary, our findings revealed that a large puncture (≥0.5 mm) remarkably suppressed the formation of pathological angiogenesis during an intravitreal injection procedure in the OIR model. This observation identified a crucial risk factor of IOP fluctuation that has been ignored in the past and affecting the neovascularization outcome in the OIR model. Moreover, our results provide a novel therapeutic perspective regarding anti-angiogenesis therapy based on the management of IOP. IOP-lowering drugs, most commonly used for reducing IOP in the clinic, are potentially a new therapeutic approach for preventing the development of retinal neovascular diseases.

## Data Availability

Data are contained within the article.

## Supplementary Materials

The following supporting information can be downloaded at: https://www.mdpi.com/article/10.3390/biom13101532/s1, Figure S1: The normal retinal vasculature after puncture; Figure S2: The IOP monitoring after intravitreal puncture.
