# Supplementary material for: HIF-1α Reduction by Lowering Intraocular Pressure Alleviated Retinal Neovascularization
Source: Biomolecules. 2023 Oct 17;13(10):1532. doi: 10.3390/biom13101532 (PMC10605289; doi:10.3390/biom13101532)
Supplement: Supplementary file 1 [file biomolecules-13-01532-s001.zip › biomolecules-2653943-supplementary.pdf]

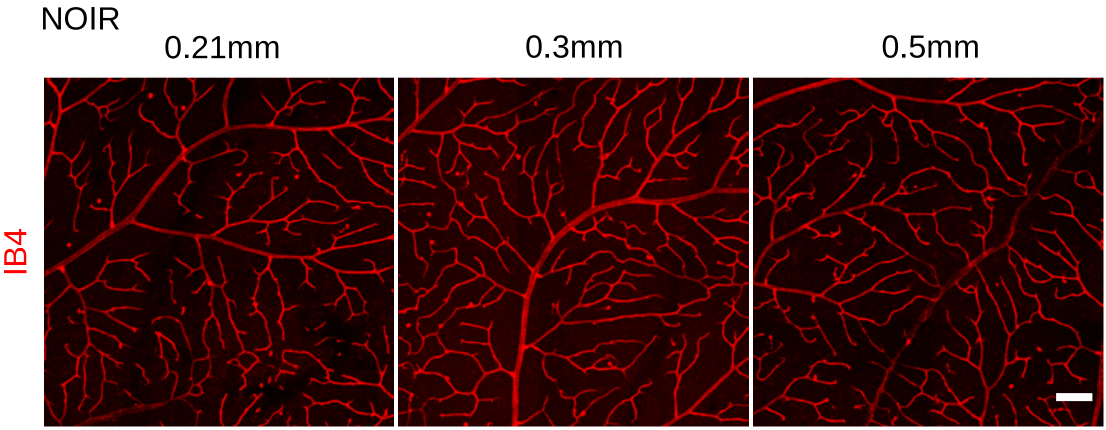

Figure S1. The normal retinal vasculature after punctures.

Representative confocal images of IB4 staining showed no detectable changes in the normal retinae after 0.5mm, 0.3mm or 0.21mm puncture. Scale bars: 100µm.

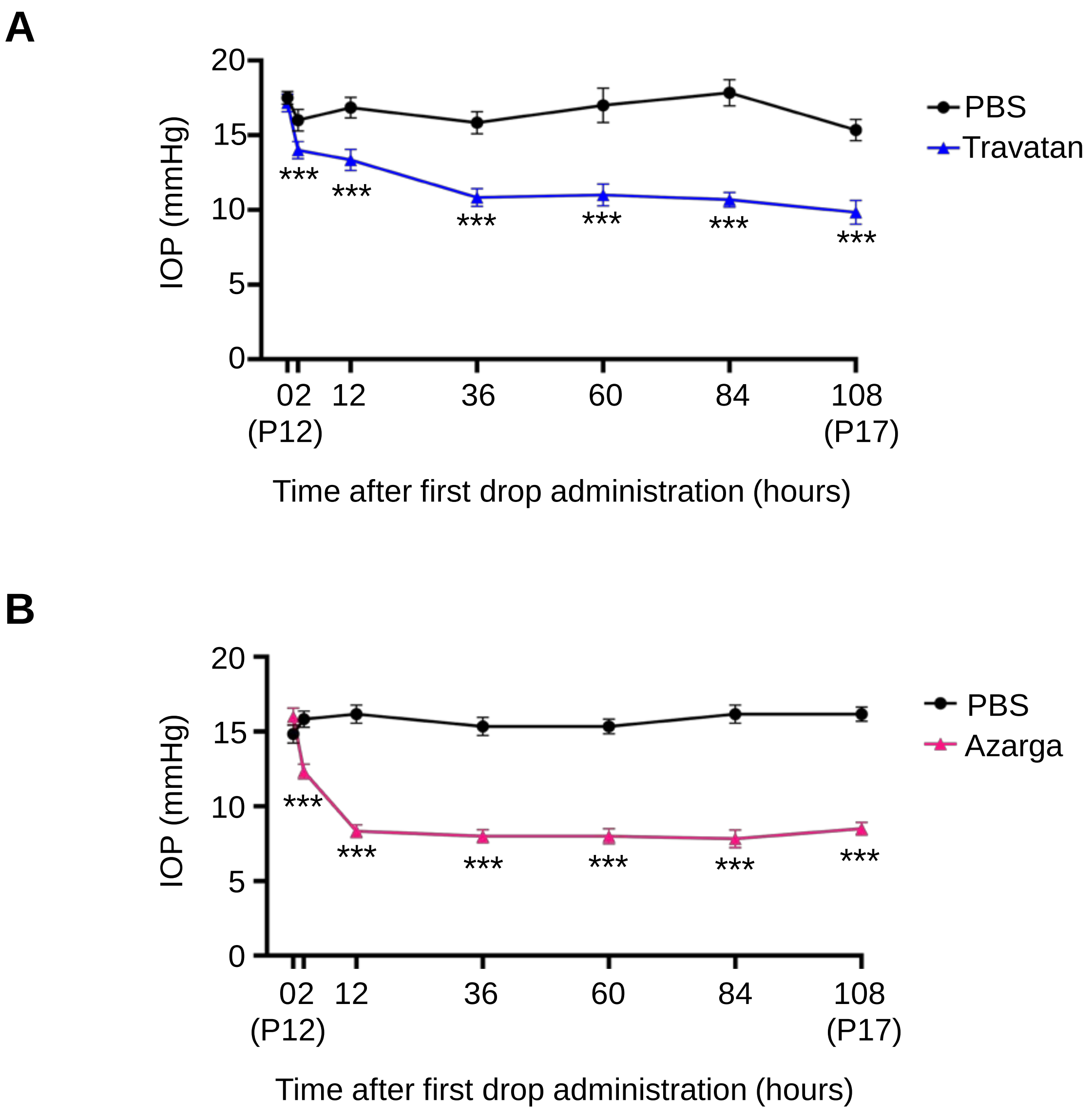

Figure S2. The IOP monitoring after intravitreal punctures.

(A) After Travatan eye drop treatment, IOP experienced a 30% reduction compared with PBS. (B) Azarga eye drop induced the reduction of IOP by 50%. (n= 6 eyes, ***P < 0.001)
